# Identification of the effects of alkalinity exposure on the gills of oriental river prawns, *Macrobrachium nipponense*

**DOI:** 10.1186/s12864-024-10659-7

**Published:** 2024-08-06

**Authors:** Shubo Jin, Rong Zhou, Xuanbin Gao, Yiwei Xiong, Wenyi Zhang, Hui Qiao, Yan Wu, Sufei Jiang, Hongtuo Fu

**Affiliations:** 1 Key Laboratory of Freshwater Fisheries and Germplasm Resources Utilization, Ministry of Agriculture and Rural Affairs, Freshwater Fisheries Research Center, Chinese Academy of Fishery Sciences, Wuxi, 214081 People’s Republic of China; 2 Wuxi Fisheries College, Nanjing Agricultural University, Wuxi, 214081 People’s Republic of China

**Keywords:** *Macrobrachium nipponense*, Alkalinity exposure, Gill, Metabolic profiling analysis, Transcriptome profiling analysis

## Abstract

*Macrobrachium nipponense* is an important commercial freshwater species in China. However, the ability of alkali tolerance of *M. nipponense* is insufficient to culture in the major saline-alkali water source in China. Thus, it is urgently needed to perform the genetic improvement of alkali tolerance in this species. In the present study, we aimed to analyse the effects of alkali treatment on gills in this species after 96 h alkalinity exposure under the alkali concentrations of 0 mmol/L, 4 mmol/L, 8 mmol/L, and 12 mmol/L through performing the histological observations, measurement of antioxidant enzymes, metabolic profiling analysis, and transcriptome profiling analysis. The results of the present study revealed that alkali treatment stimulated the contents of malondialdehyde, glutathione, glutathione peroxidase in gills, indicating these antioxidant enzymes plays essential roles in the protection of body from the damage, caused by the alkali treatment. In addition, high concentration of alkali treatment (> 8 mmol/L) resulted in the damage of gill membrane and haemolymph vessel, affecting the normal respiratory function of gill. Metabolic profiling analysis revealed that Metabolic pathways, Biosynthesis of secondary metabolites, Biosynthesis of plant secondary metabolites, Microbial metabolism in diverse environments, Biosynthesis of amino acids were identified as the main enriched metabolic pathways of differentially expressed metabolites, which are consistent with the previous publications, treated by the various environmental factors. Transcriptome profiling analyses revealed that the alkali concentration of 12 mmol/L has more regulatory effects on the changes of gene expression than the other alkali concentrations. KEGG analysis revealed that Phagosome, Lysosome, Glycolysis/Gluconeogenesis, Purine Metabolism, Amino sugar and nucleotide sugar metabolism, and Endocytosis were identified as the main enriched metabolic pathways in the present study, predicting these metabolic pathways may be involved in the adaption of alkali treatment in *M. nipponense*. Phagosome, Lysosome, Purine Metabolism, and Endocytosis are immune-related metabolic pathways, while Glycolysis/Gluconeogenesis, and Amino sugar and nucleotide sugar metabolism are energy metabolism-related metabolic pathways. Quantitative PCR analyses of differentially expressed genes (DEGs) verified the accuracy of the RNA-Seq. Alkali treatment significantly stimulated the expressions of DEGs from the metabolic pathways of Phagosome and Lysosome, suggesting Phagosome and Lysosome play essential roles in the regulation of alkali tolerance in this species, as well as the genes from these metabolic pathways. The present study identified the effects of alkali treatment on gills, providing valuable evidences for the genetic improvement of alkali tolerance in *M. nipponense*.

## Introduction

Oriental river prawn, *Macrobrachium nipponense*, is an important commercial freshwater species, which is widely distributed in China and the other Asian counties [1]. The annual production of this species is over 200 thousand thons in China, accounting for 5.72% of the total production of freshwater prawns, and producing huge economic benefits. The main regions for the *M. nipponense* culture included Jiangsu province, Anhui province, Zhejiang province and Jiangxi province, while the productions in the northeast and northwest part of China were limited [2]. A reasonable explanation for this is that the water resources in the northeast and northwest regions of China are mainly saline-alkali water, which has negative effects on the culture of *M. nipponense* in these regions.

The abilities of alkali tolerance have been identified in many fish and crustacean species. The *LC*_*50*_ values of 24 h of alkali treatment in *Ctenopharyngodon idellus* (pH 8.65), *Hypophthalmichthys molitrix* (pH 8.74), and *Aristichthys nobolis* (pH 9.14) were 82.2 mmol/L, 95 mmol/L, and 65.7 mmol/L, respectively [3]. Chi et al. revealed that the *LC*_*50*_ values of *Tribolodon brandti Dybowski* were 98.79 mmol/L at 12 h, 89.31 mmol/L at 24 h, 79.34 mmol/L at 48 h, 78.45 mmol/L at 72 h and 68.44 mmol/L at 96 h, and the safe alkali concentration was identified as 18.79mmol/L, under the condition of pH 8.5 ± 0.5 and NaCl of 3.1–7.4 g/L [4]. *Gymnocypris przewalskii* can be well adapted under the alkalinity value of 64 mmol/L [5]. The abilities of alkali tolerance in fish were dramatically stronger than those of crustacean species. The *LC*_*50*_ value of 24 h of *Penaeus vannamei* was 12.40 mmol/L, and the safe alkali concentration was only 2.77 mmol/L under the pH of 8.11–8.72 [6]. The *LC*_*50*_ values of 24 h of *Penaeus chinensis* were 22.00 mmol/L, 11.66 mmol/L, 6.57 mmol/L, and 3.28 mmol/L, respectively, under the pH of 8.6, 9.0, 9.3, and 9.5 [7]. *Palaemon przewalskii* can be only normally bred and grow under the alkalinity value of < 3.5 mmol/L [8]. Ren et al. identified that the *LC*_*50*_ values of 12 h, 24 h, 48 h, 72 h, and 96 h were 27.66 mmol/L, 26.94 mmol/L, 22.51 mmol/L, 15.00 mmol/L and 14.42 mmol/L, respectively, with safety value of 4.71 mmol/L under conditions of water temperature of (23.1 ± 1.48) ℃, pH= (8.9 ± 0.30), salinity of (0.62 ± 0.27), dissolved oxygen level of (7.2 ± 0.30) mg/L, using the juvenile Taihu No2 as the research species (new variety of *M. nipponense* through genetic breeding) [9]. The freshwater resource is limited in China. Thus, the long-term goal of *M. nipponense* industry is to culture *M. nipponense* in the saline-alkali water resource, which may play essential roles in promoting the annual production and maintaining the sustainable development of *M. nipponense*. *M. nipponense* showed stronger abilities of alkali tolerance, compared to those of the other crustacean species, indicating this species has the potential possibility to culture under the saline-alkali water resource. However, the current abilities of alkali tolerance are insufficient to culture under the main saline-alkali water resource in China. Thus, it is urgently needed to carry out the mechanism of alkalinity tolerance in *M. nipponense*, including the identification of alkali-tolerance related genes and SNPs, promoting the genetic improvement of alkali tolerance, in order to maintain the sustainable development of this species.

The alkali-tolerance related genes have been selected in *Lateolabrax maculatus* [10], *Luciobarbus capito* [11], *Leuciscus waleckii* [12, 13] through performing the transcriptome profiling analyses. These studies suggested that stress response and extreme environment adaptation related pathways were identified as the main enriched metabolic pathways of differentially expressed genes, including phenylalanine, tyrosine and tryptophan biosynthesis, cell cycle and DNA replication.

Gill is the main organ for respiration in aquatic animals. The blood vessels are covered on the surface of the gill filaments, and oxygen in the water enters the blood through these vessels for respiratory function. The present study was aimed to carry out the effects of alkalinity exposure on antioxidant status, metabolic function, and immune response in the gill of *M. nipponense*. In the present study, the effects of alkalinity on the morphology and the activities of antioxidant enzymes in gills of *M. nipponense* were analysed after the 96 h treatment under the different alkali concentrations. Furthermore, the genes and metabolites were selected from gills through performing the integrated analysis of transcriptome and metabolome, which are involved in the response of alkali treatment in this species. The present study will promote the genetic improvement of alkali tolerance in this species.

## Materials and methods

### Sample collection

The wild *M. nipponense* population from Yangtze River in the present study were provided by Dapu *M. nipponense* Breeding Base in Wuxi, China (120°13′44″E, 31°28′ 22″N) and collected in June 2023. The prawns were stayed in aerated freshwater with dissolved oxygen content of ≥ 6 mg/L for 3 days in prior to the alkali treatment. A total of 1,200 *M. nipponense* prawns were collected for alkali treatment. The body weight for male prawns were 3.79–4.21 g, and the body weight for female prawns were 2.31–3.14 g. All prawns were randomly divided into four alkali concentrations. Each alkali concentration prepared three tanks, and each tank contained 100 prawns. Previous study has identified that LC50 values of alkalinity exposure in *M. nipponense* was 14.42 mmol/L at 96 h [9]. Thus, NaHCO_3_ was added into the aerated freshwater to prepare the water environment with alkalinity, and four alkali concentrations were prepared, including 0 mmol/L (control, water with NaHCO_3_), 4 mmol/L, 8 mmol/L and 12 mmol/L under conditions of water temperature of (28.3 ± 1.26) ℃, pH = (7.81–8.32), and dissolved oxygen level of > 6.0 mg/L. The alkali concentrations were measured, according to the criterion of SC/T9406-2012. All prawns were cultured in the water environment with different alkali concentrations for 96 h. Gills were collected from each alkali concentrations, and used for the analyses of morphological changes, measurement of antioxidant enzymes, metabolic profiling analysis, transcriptome profiling analysis, and qPCR analysis. Five gills were collected from each alkali concentration, and pooled at together to form a biological replicate. Three biological replicates were prepared for the measurement of antioxidant enzymes, transcriptome profiling analysis and qPCR analysis, while eight biological replicates were prepared for the metabolic profiling analysis.

### Measurement of the activities of antioxidant enzymes

The commercial kits were used to measure the activities of malondialdehyde (MDA), superoxide dismutase (SOD), catalase (CAT), glutathione (GSH), glutathione peroxidase (GSH-PX), and total antioxidant capacity (T-AOC) in gills, purchased from the Nanjing Jiancheng Bioengineering Institute, following the manufacturer’s instructions. All the antioxidant indexes were measured by using microplate reader (Bio-rad iMark, San Francisco, America), followed the manufacturer’s instructions.

### Haematoxylin and eosin (HE) staining of gills

The gill tissues (*N* = 3) from each alkali concentration were fixed in 4% paraformaldehyde, which were used for the observations of morphological changes in gills, caused by the alkali treatment. Previous publications have been well described the detailed procedures of HE staining [14, 15]. Briefly, varying ethanol concentrations were used to dehydrate the gill samples. The dehydrated gill samples were then transparent and embedded by using different percentage of xylene/wax mixture. Finally, the embedded gill samples were sliced to 5 μm thickness by using a slicer (Leica, Wetzlar, Germany). The slices were stained by HE for 3–8 min. Olympus SZX16 microscope (Olympus Corporation, Tokyo, Japan) was used to view the morphological changes of gills, caused by the alkali treatment.

### Metabolic profiling analysis

The differentially expressed metabolites (DEMs) in the gills of *M. nipponense*, caused by the alkali treatment, were selected through performing the metabolic profiling analysis. Liquid chromatography-mass spectrometry (LC/MS) analyses were used to determine the metabolic profiling analysis [16]. Previous publications have been well described the detailed procedures for the metabolic profiling analysis [17]. An ACQUITY UHPLC system (Waters Corporation, Milford, USA) and an AB SCIEX Triple TOF 5600 System (AB SCIEX, Framingham, MA) were employed to analyse the metabolic profiling in both ESI positive and ESI negative ion modes. The robustness and predictive ability of the model was measured by the criterion of a seven-fold cross-validation. The permutation tests were further employed to perform the validation.

### Transcriptome profiling analysis

The differentially expressed genes (DEGs) in the gills of *M. nipponense*, caused by the alkali treatment, were selected through performing the transcriptome profiling analysis. Previous publications have been well described the detailed procedures for the RNA-Seq and analysis [18, 19]. Briefly, the total RNA was extracted by using RNAiso Plus Reagent (TaKaRa), according to the manufacturer’s instructions. RNA integrity number (RIN) value of > 7.0 indicated the integrity of total RNA, measured by using A 2100 Bioanalyzer (Agilent Technologies, Inc.). RNA concentrations were measured by using ultraviolet spectrophotometer (Eppendorf, Germany). A total of 4 µg of total RNA was used to construct the library. Illumina Hiseq-2500 sequencing platform was employed to perform the transcriptome profiling analysis under the parameter of PE150.

Fastp software was employed to remove the low-quality raw reads with the default parameters [20]. The HISAT2 software was then employed to map the obtained clean reads to the *M. nipponense* reference genome (Genbank access numbers: GCA_015104395.2) [21]. Genes were annotated in the Gene Ontology (GO) (http://www.geneontology.org/, accessed on 15 August 2023) [22], Cluster of Orthologous Groups (COG) (http://www.ncbi.nlm.nih.gov/COG/, accessed on 15 August 2023) [23], and Kyoto Encyclopedia of Genes and Genomes (KEGG) databases (http://www.genome.jp/kegg/, accessed on 15 August 2023) [24], using an E-value of 10^− 5^ [18]. Gene expression was calculated using the FPKM method, where FPKM = cDNA fragments/mapped fragments (millions)/transcript length (kb), using HTSeq-count [25]. DESeq2 was used to perform the differential expression analysis [26]. The Benjamini–Hochberg correction method was used to calculate the false discovery rate (FDR) [27] with q-value < 0.05. Fold change > 2 was considered as the up-regulated differentially expressed genes (DEGs), and fold change < 0.5 was considered as the down-regulated DEGs.

### Correlation analysis of metabolome and transcriptome analysis

The integrated analysis of metabolome and transcriptome sequencing data was performed to reveal the post-transcriptional regulatory status of genes by using the Spearman method. Euclidean distance matrix was calculated to cluster differential metabolites and genes by complete linkage method.

### qPCR analysis

The expressions of DEGs were verified by qPCR analyses in the present study. The detailed procedures have been well described in the previous publications [28, 29]. Briefly, total RNA of each sample was extracted by using the UNlQ-10 Column Trizol Total RNA Isolation Kit (Sangon, Shanghai, China). The total RNA quality was measured by 1.2% agarose gel electrophoresis, and RNA concentrations were measured by using ultraviolet spectrophotometer (Eppendorf, Germany). The cDNA template was synthesized from 1 µg total RNA of each sample by using PrimeScript™ RT reagent kit (Takara Bio Inc., Japan), according to the manufacturer’s instructions. The expression levels were measured by using the UltraSYBR Mixture (CWBIO, Beijing, China), and conducted on the Bio-Rad iCycler iQ5 Real-Time PCR System (Bio-Rad), according to the reaction system of manufacturer’s instructions. All primers used for qPCR analysis were listed in Table 1. The previous publication has been identified the eukaryotic translation initiation factor 5 A (EIF) as the suitable and stable reference gene for qPCR analysis in *M. nipponense* [30]. The 2^−ΔΔCT^ method was used to determine the relative expression levels [31].

**Table 1:** Primers used in the present study

Gene	Forward	Reverse
LAMP1	GGGAATCCGGCAAACACAAC	GGGATGTCACTGGGTCGAAG
CysP4	TGGGTCATTGGAAGGTCAGC	CGTGTCAATGCCCTTGTTGG
alphaNAGAL	ACCCCAACAAGTGGCAATGA	GTTCGTAGAGGTCGGACACG
Cathepsin B	ATTCCCGAATGCGAGCATCA	CCTCAACGGGGCCATTAGTC
Cathepsin L	TGACGTTTGCCTGAGTCGTT	TTCGAGCCAACCCATTCTCC
PK	CAGTCCTTGCAGCACTACGA	CTCTGATGAGCCAGTCGTCC
PEPCK	GGGCCCTCGCTAACAGAAAT	TCGTTCACTCCCATCGCAAA
FBP1	GACTACCCAGACTGCCATCG	CTTGAACGTTGACACTGCCG
RGC	ACCACTACATCTGTGCAGGC	GGTGTAACAAAGCACCAGCG
RRL	GCAGAGAAGCTTGGCCCATA	GCAAGCAGAAGAGAGTTGCG
PC	GAAATTTCCCTCCCGGCTGA	GAGGACTGGTCTTGTTGCCA
galU	ACCACGACCCTTACAACACC	CATCCTAGGTACCGGTGGGA
Phosphoglucomutase	TATCCGAGAGAAGGACGGCA	TGCAAGGGTCTGCTTCACAA
HSP70	GAGTCTTCGGTGGCAGACAA	CTGAACCCTGCTGACCTGAG
Dab	ATACGTGCGAGTGTCATGCT	GCATTAACCGTCACTTCCGC
E3-Su(dx)	TTCAGCCTGATTCGCAAGGT	AAGGTGGATAACAGCCAGCC

### Statistical analysis

All of the statistical analyses in the present study were measured by SPSS 23.0, estimated by the Duncan’s multiple range test in one-way ANOVA. Quantitative data were expressed as the mean ± SD. *P* < 0.05 indicated significant difference. The homogeneity of variances was measured in prior to ANOVA (Sig.>0.05).

## Results

### Measurement of antioxidant enzymes under the treatment of alkali environment

**Fig. 1:**
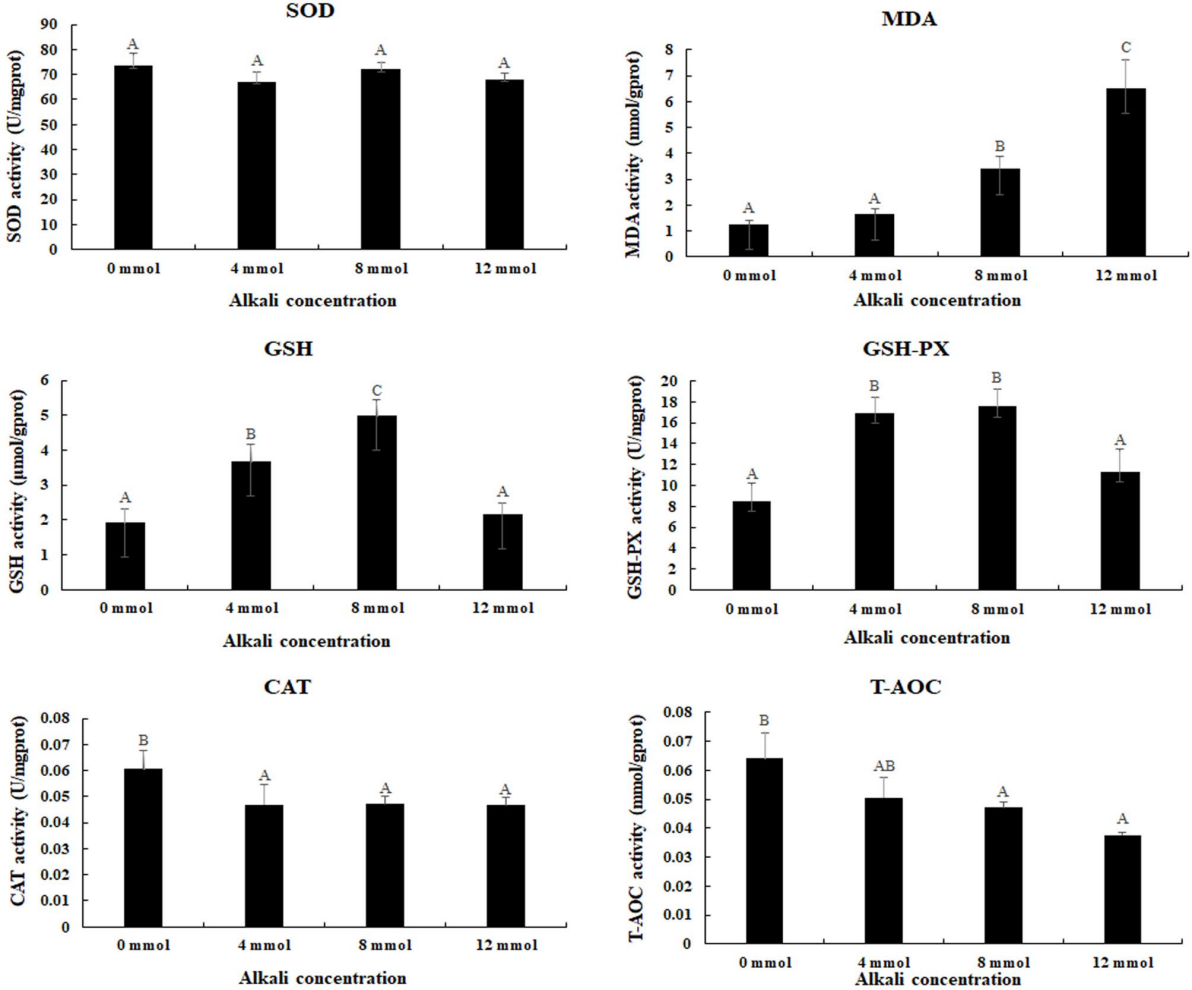
The measurements of the activities of antioxidant enzymes in the gills under the treatment of different alkali concentrations. CAT: Catalase; GSH: Glutathione; GSH-PX: Glutathione peroxidase; MDA: malondialdehyde; SOD: Superoxide dismutase; T-AOC: Total antioxidant capacity. Data are shown as mean ± SD (standard deviation) of tissues from three biological replicates. Capital letters indicated the significant difference of the activities of antioxidant enzymes between different alkali concentrations

The activities of antioxidant enzymes were measured under the treatment of different alkali concentrations (Fig. 1). The MDA content gradually increased with the increase of alkali concentration, and reached the peak at the alkali concentration of 12 mmol/L (*P* < 0.05), while CAT and T-AOC showed the highest activities at the alkali concentration of 0 mmol/L, and gradually decreased with the increase of alkali concentrations (*P* < 0.05). The activities of GSH and GSH-PX gradually increased, and reached the peak at the alkali concentration of 8 mmol/L (*P* < 0.05), and then decreased. However, the activities of SOD showed no differ between different alkali concentrations (*P* > 0.05).

### Morphological changes of gills under the treatment of alkali environment

**Fig. 2:**
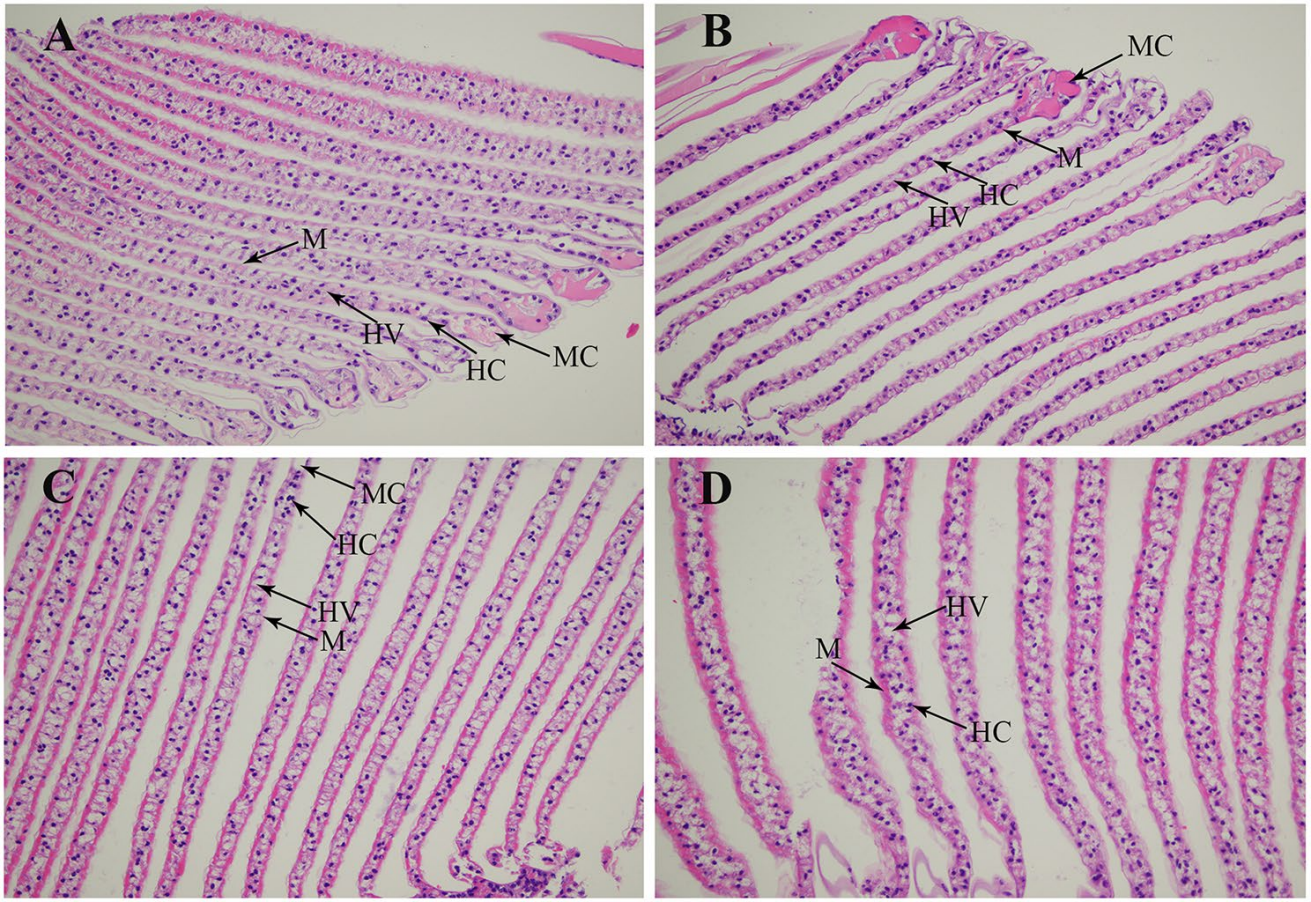
The morphological changes of gills under the treatment of different alkali concentrations by histological observations. HC: hemocytes; HV: hemolymph vessel; M: membrane; MC: marginal channel. Scale bars = 20 μm. (**A**): the histological observation of gills under the alkali concentration of 0 mmol/L; (**B**): the histological observation of gills under the alkali concentration of 4 mmol/L; (**C**): the histological observation of gills under the alkali concentration of 8 mmol/L; (**D**): the histological observation of gills under the alkali concentration of 12 mmol/L

The morphological changes of gills were analysed through performing the haematoxylin and eosin (HE) staining after the treatment of different alkali concentrations (Fig. 2). The histological observations revealed that the normal gill of *M. nipponense* included marginal channel, hemocytes, hemolymph vessel, and membrane. The gills showed normal morphological structures under the alkali concentrations of 0 mmol/L and 4 mmol/L, and no significant damages were observed. The membrane of gill became thinner, and cannot support the normal morphological structure of gill when the alkali concentration reached to 8 mmol/L and 12 mmol/L. Thus, hemolymph vessel were increased, affecting the normal respiratory function of gill [32, 33].

### Metabolic profiling analysis of gills under the treatment of alkali environment

**Fig. 3:**
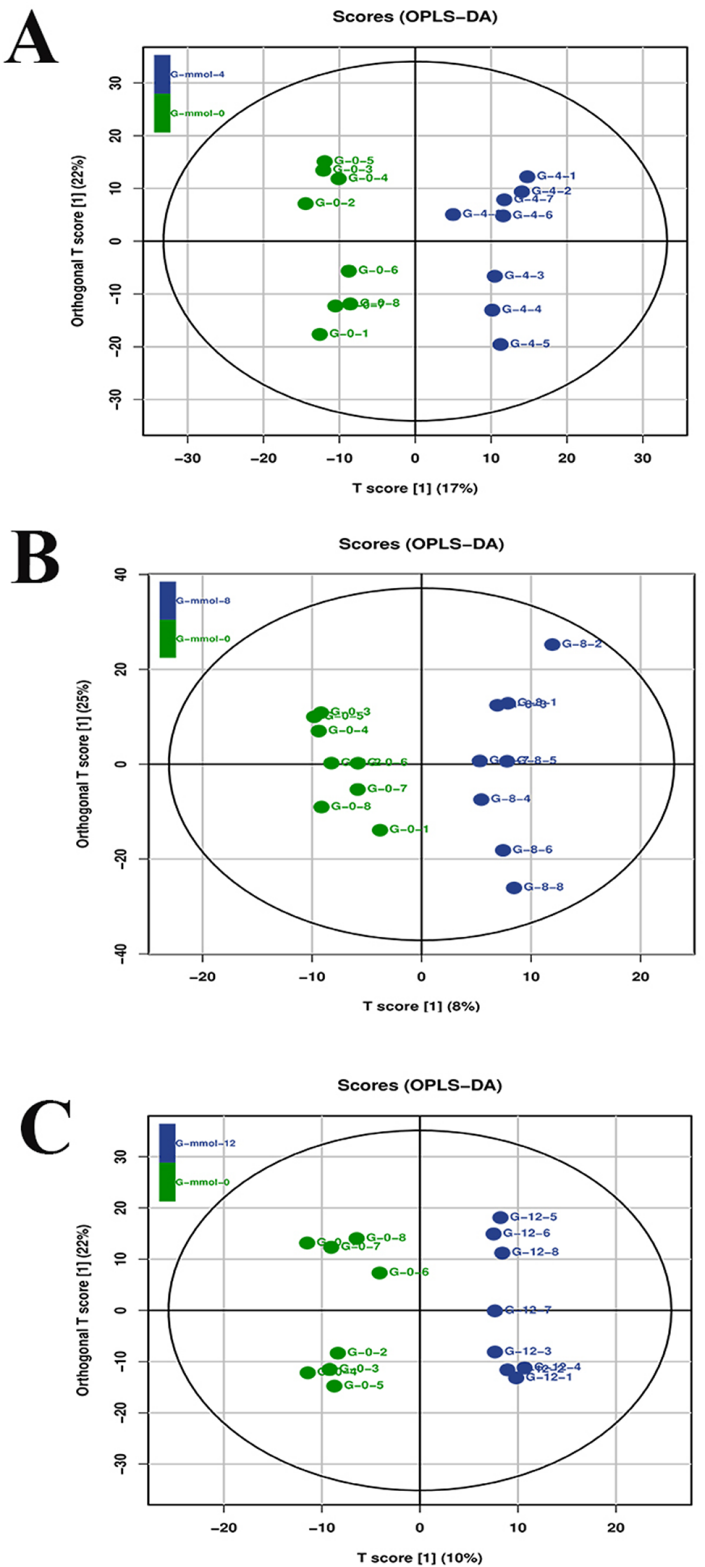
Orthogonal projections to latent structures discriminate analysis (OPLS-DA) analysis of gills after the treatment of different alkali concentrations. The LC-MS spectra was used to measure the OPLS-DA score. (**A**) indicated the OPLS-DA analysis between 0 mmol/L vs. 4 mmol/L; (**B**) indicated the OPLS-DA analysis between 0 mmol/L vs. 8 mmol/L; (**C**) indicated the OPLS-DA analysis between 0 mmol/L vs. 12 mmol/L

The overall quality of the metabolic profiling analysis was analysed by the latent structures discriminant analysis in the present study (Fig. 3). It suggested a robust and reliable model to identify the different metabolic patterns in the gills of *M. nipponense* after the treatment of different alkali concentrations.

The differentially expressed metabolites (DEMs) were selected in the gills of *M. nipponense* after the treatment of different alkali concentrations through performing the LC/MS analysis, using the criterion of > 2.0 as the up-regulated metabolite and < 0.5 as the down-regulated metabolite. A total of 171 (77 up-regulated metabolites and 94 down-regulated metabolites), 37 (15 up-regulated metabolites and 22 down-regulated metabolites), 87 (52 up-regulated metabolites and 35 down-regulated metabolites) DEMs were identified between the comparison of 0 mmol/L vs. 4 mmol/L, 0 mmol/L vs. 8 mmol/L, and 0 mmol/L vs. 12 mmol/L, respectively. Metabolic pathways, Biosynthesis of secondary metabolites, Biosynthesis of plant secondary metabolites, Microbial metabolism in diverse environments, Biosynthesis of amino acids were identified as the main enriched metabolic pathways of DEMs among all of these three comparisons.

### Transcriptome profiling analysis of gills under the treatment of alkali environment

A total of 44,071 genes were highly matched the known genes in the *M. nipponense* genome, which is almost consistent with the number of genes (44,086) in the *M. nipponense* genome. In addition, the present study also predicted 4,134 novel isoforms. The regulatory functions of these genes need further investigated.

The differentially expressed genes (DEGs) were selected from gills under the treatment of alkali environment through performing the transcriptome profiling analysis, using the criterion of > 2.0 as the up-regulated gene and < 0.5 as the down-regulated gene. A total of 1,107 DEGs were selected between the alkali concentration of 0 mmol/L and 4 mmol/L, of which 301 genes were up-regulated and 806 genes were down-regulated. A total of 1,181 DEGs were identified between the alkali concentration of 4 mmol/L and 8 mmol/L, including 880 up-regulated genes and 301 down-regulated genes. A total of 1,903 genes were differentially expressed between the alkali concentration of 8 mmol/L and 12 mmol/L, including 1,419 up-regulated genes and 484 down-regulated genes. In addition, the number of DEGs between 0 mmol/L vs. 4 mmol/L, 0 mmol/L vs. 8 mmol/L, and 0 mmol/L vs. 12 mmol/L were 1,107, 177, and 3,294, respectively.

**Fig. 4:**
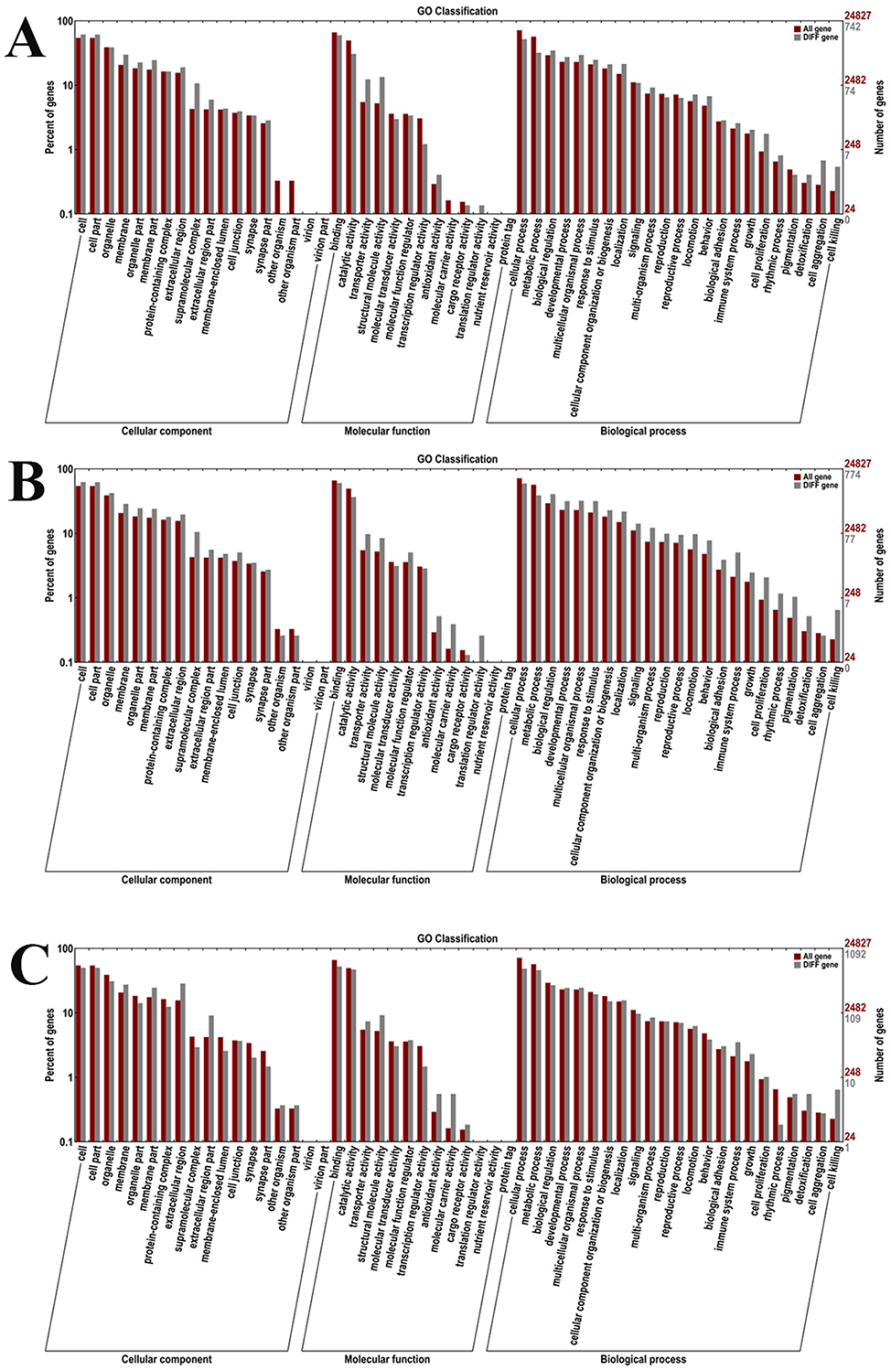
Gene Ontology (GO) analysis of DEGs after the treatment of different alkali concentrations in gills. (**A**): GO analysis between 0 mmol/L vs. 4 mmol/L; (**B**): GO analysis between 4 mmol/L vs. 8 mmol/L; (**C**): GO analysis between 8 mmol/L vs. 12 mmol/L

The number of DEGs between the comparison of 0 mmol/L vs. 4 mmol/L, 4 mmol/L vs. 8 mmol/L, and 8 mmol/L vs. 12 mmol/L were 834, 839, and 1,134, respectively, which were annotated in GO database. Cell, Cell part, Binding, Cellular process, and Organelle represented the main enriched functional groups in all of these three comparisons (Fig. 4).

**Fig. 5:**
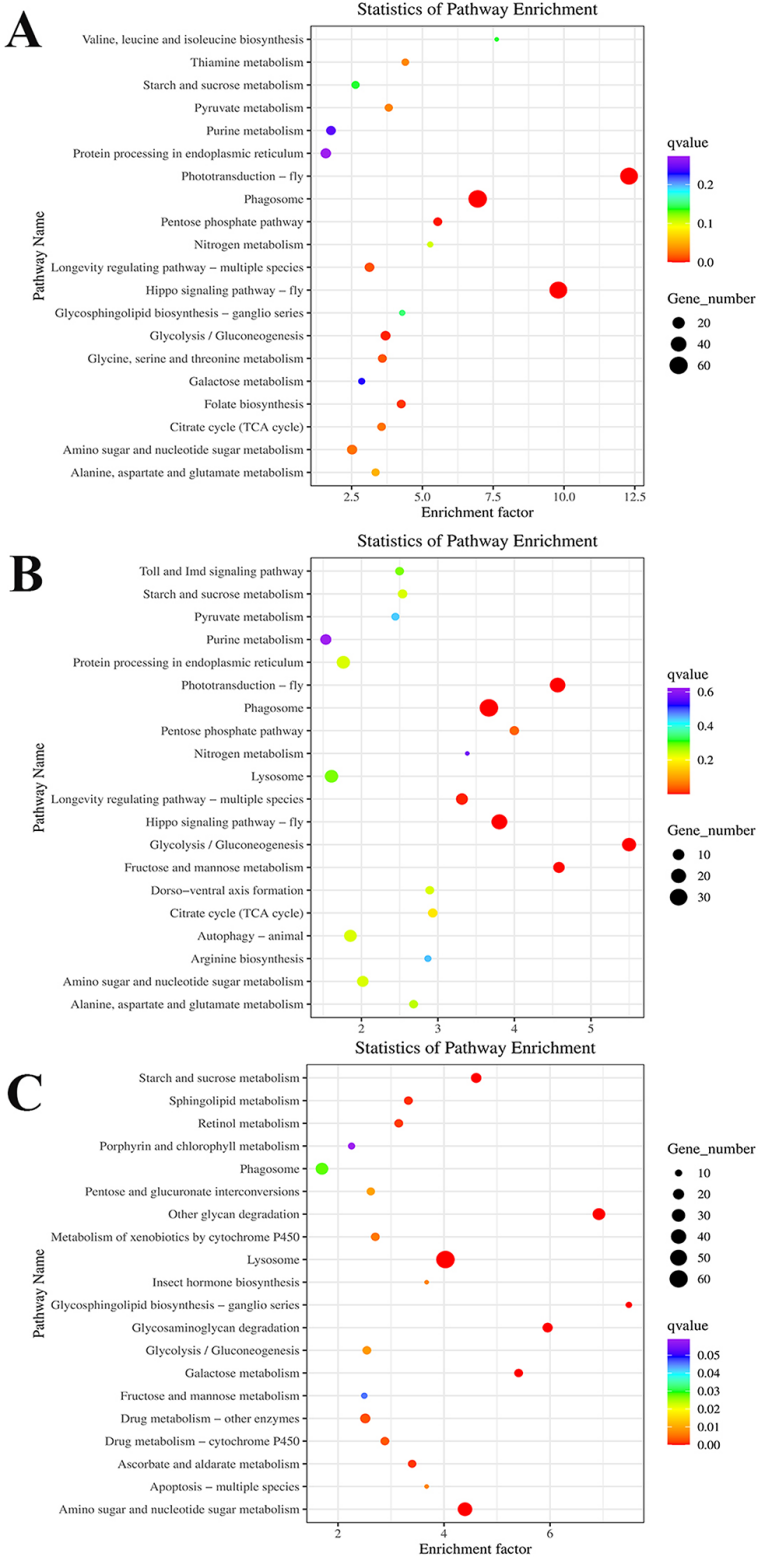
Kyoto Encyclopedia of Genes and Genomes (KEGG) analysis of DEGs after the treatment of different alkali concentrations in gills. (**A**): KEGG analysis between 0 mmol/L vs. 4 mmol/L; (**B**): KEGG analysis between 4 mmol/L vs. 8 mmol/L; (**C**): KEGG analysis between 8 mmol/L vs. 12 mmol/L

The number of DEGs between the comparison of 0 mmol/L vs. 4 mmol/L, 4 mmol/L vs. 8 mmol/L, and 8 mmol/L vs. 12 mmol/L were 235, 217, and 424, respectively, which were annotated in KEGG database. Phagosome, Lysosome, Glycolysis/Gluconeogenesis, Purine Metabolism, Amino sugar and nucleotide sugar metabolism, and Endocytosis represented the main enriched metabolic pathways in all of these three comparisons (Fig. 5).

A total of 19 genes were selected from these metabolic pathways, of which alkali treatment leads to the significant changes of gene expression in *M. nipponense*. All of these genes were differentially expressed in at least two comparisons, including six genes, which were differentially expressed among all of these three comparisons. These selected DEGs were considered as the key genes in *M. nipponense*, which play essential roles in the protection of body from damage, caused by the alkali treatment in *M. nipponense* (Table 2).

**Table 2:** The DEGs selected from the present study

Gene	Accession number	Species	Metabolic pathways	Fold change		
				0 vs. 4	4 vs. 8	8 vs. 12
Actin-2, muscle-specific-like	XP_027223005.1	*Penaeus vannamei*	Phagosome	-9.17	8.76	-5.06
Actin	XP_027224003.1	*Penaeus vannamei*	Phagosome	-9.62	10.21	-4.2
Lysosome-associated membrane glycoprotein 1 (LAMP1)	XP_027215562.1	*Penaeus vannamei*	Phagosome; Lysosome		15.79	17.26
Cysteine proteinase 4 (CysP4)	XP_027216087.1	*Penaeus vannamei*	Phagosome; Lysosome		158.12	17.15
Cathepsin L	AHW49157.1	*Macrobrachium rosenbergii*	Phagosome; Lysosome		11.11	7.49
alpha-N-acetylgalactosaminidase (alphaNAGAL)	XP_027217853.1	*Penaeus vannamei*	Lysosome		4.47	5.65
Cathepsin B	AUG69383.1	*Macrobrachium rosenbergii*	Lysosome		19.83	8.28
Cathepsin S	KAI2655169.1	*Labeo rohita*	Lysosome		9.03	7.56
Pyruvate kinase (PK)	APP91606.1	*Macrobrachium nipponense*	Glycolysis/Gluconeogenesis; Purine metabolism	-2.56	3.13	-2.49
Phosphoenolpyruvate carboxykinase (PEPCK)	XP*_*027234775.1	*Penaeus vannamei*	Glycolysis/Gluconeogenesis	-4.9	5.01	2.1
Fructose-1,6-bisphosphatase 1 (FBP1)	XP_027236390.1	*Penaeus vannamei*	Glycolysis/Gluconeogenesis	-14.91	4.86	-2.23
Receptor-type guanylate cyclase (RGC)			Purine metabolism		98.01	-6.19
Ribonucleoside-diphosphate reductase large subunit (RRL)	XP_027233205.1	*Penaeus vannamei*	Purine metabolism		10.55	-9.42
Putative chitinase (PC)	AWU67222.1	*Crangon crangon*	Amino sugar and nucleotide	-7.16	7.06	
UTP–glucose-1-phosphate uridylyltransferase (galU)	XP_027217969.1	*Penaeus vannamei*	Amino sugar and nucleotide	-4.79	3.44	
Phosphoglucomutase	XP_047482274.1	*Penaeus chinensis*	Amino sugar and nucleotide		4.21	-3.75
Heat shock protein 70 (HSP70)	ANQ44703.1	*Bythograea thermydron*	Endocytosis	-3.75	8.72	-3.03
Disabled protein (Dab)	XP_027217927	*Penaeus vannamei*	Endocytosis	-3.31	2.85	
E3 ubiquitin-protein ligase Su(dx) (E3-Su(dx))	XP_027232977.1	*Penaeus vannamei*	Endocytosis		-2.31	2.61

### Correlation analysis of DEMs and DEGs

**Fig. 6:**
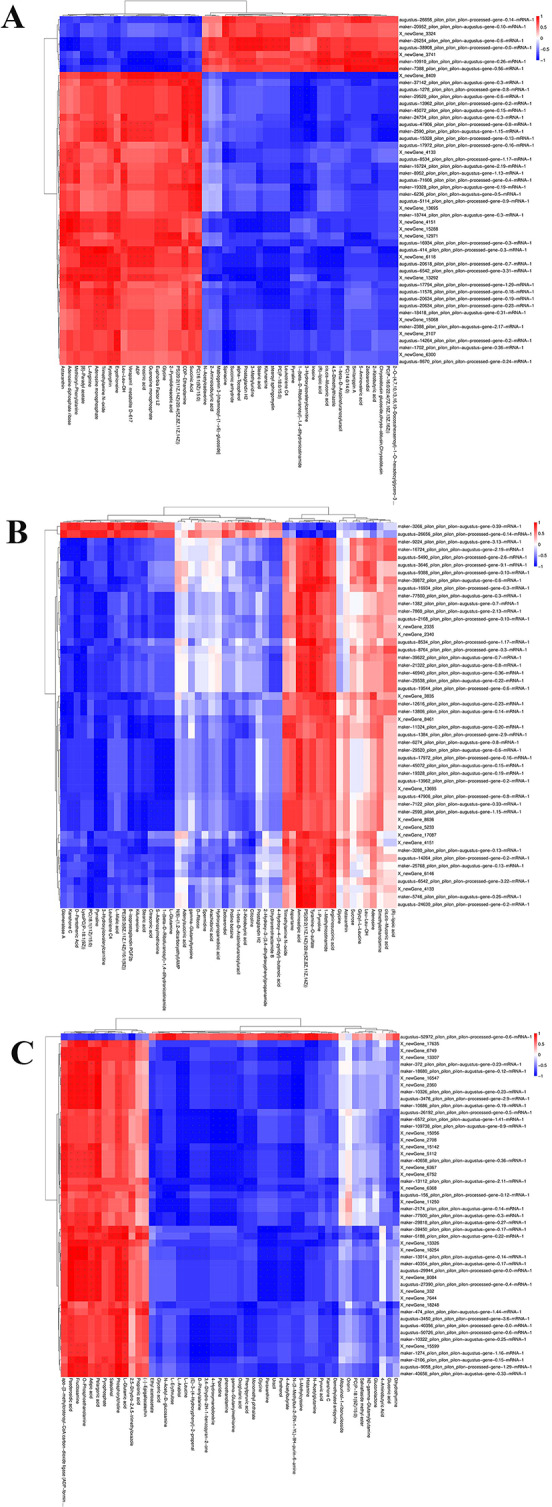
Clustered heat map of correlation analysis of DEGs and DEMs after the treatment of different alkali concentrations in *M. nipponense*. The clustered heat map with genes in columns and metabolites in rows to show the connection between genes and metabolites. The positively and negative correlation among transcriptomics and metabolomics were showed by the red and blue, respectively. (**A**): Correlation analysis of DEGs and DEMs between 0 mmol/L vs. 4 mmol/L; (**B**): Correlation analysis of DEGs and DEMs between 4 mmol/L vs. 8 mmol/L; (**C**): Correlation analysis of DEGs and DEMs between 8 mmol/L vs. 12 mmol/L

The correlation between DEGs and SDMs was calculated by using the spearman algorithm. The significant DEMs and DEGs were analysed by correlation hierarchical clustering, and showed in Fig. 6. The strong correlation between each DEGs and DEMs will further highlight the association of each transcript with a specific metabolite.

### qPCR verification

**Fig. 7:**
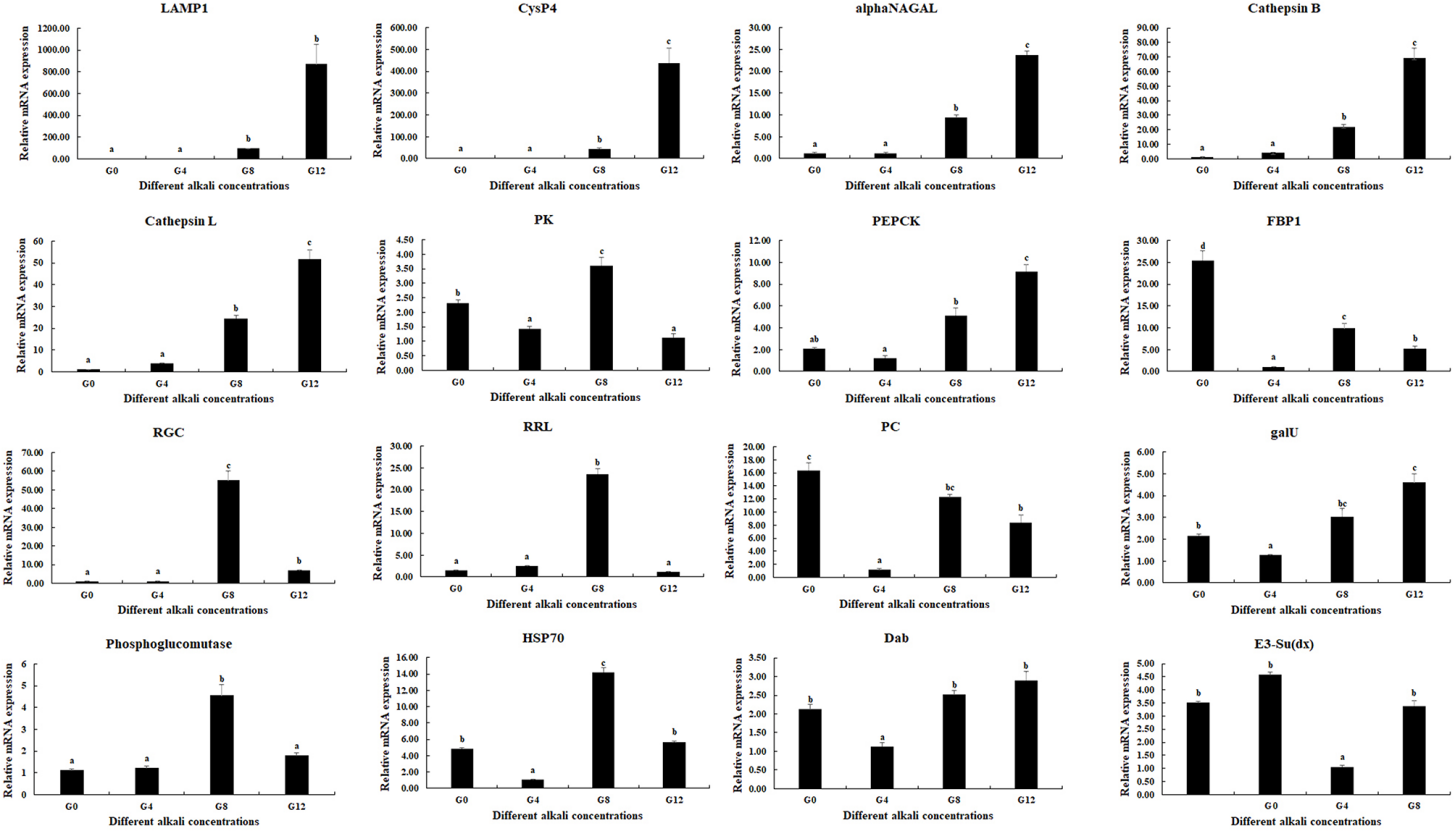
qPCR analyses of the expressions of DEGs in the gills under the treatment of different alkali concentrations. Data are shown as mean ± SD (standard deviation) of tissues from three biological replicates. Letters indicated the significant difference of the expressions of DEGs between different alkali concentrations

qPCR was used to verify the expressions of candidate DEGs, which were selected and predicted to be involved in the process of alkali tolerance in the present study (Fig. 7). qPCR analyses generally showed the same expression pattern with those of RNA-Seq. Five DEGs from the metabolic pathways of Phagosome and Lysosome were gradually increased with the increase of alkali concentrations, and reached the peak at the alkali concentration of 12 mmol/L (*P* < 0.05), while the expressions between 0 mmol/L and 4 mmol/L showed no differ (*P* > 0.05). The other 11 genes showed fluctuant expression pattern, of which three genes showed the highest expressions at the alkali concentrations of 12 mmol/L (*P* < 0.05), including Phosphoenolpyruvate carboxykinase (*PEPCK*), UTP–glucose-1-phosphate uridylyltransferase (*galU*), and Disabled protein (*Dab*). The expressions of five DEGs reached the peak at the alkali concentrations of 8 mmol/L, and showed significant difference with those of the other concentrations (*P* < 0.05), including Pyruvate kinase (*PK*), Receptor-type guanylate cyclase (*RGC*), Ribonucleoside-diphosphate reductase large subunit (*RRL*), Phosphoglucomutase, and Heat shock protein 70 (*HSP70*).

## Discussion

Previous study identified that *M. nipponense* showed stronger abilities to resist the alkali treatment than the other crustacean species, while showed lower abilities than those of fish species [9]. There is wild saline-alkali water resource in the northeast and northwest regions of China. The long-term goal of *M*,* nipponense* industry is to culture the prawns in the saline-alkali water resources, playing essential roles in the increase of the annual production of this species. Thus, genetic improvement is need to improve the abilities of alkali tolerance in this species, in order to maintain the sustainable development of *M. nipponense*. In the present study, the effects of alkali treatment on the gills of *M. nipponense* were carried out, including the identification of the genes and metabolites, involved in the response of alkali treatment. This study will provide valuable evidences for the genetic improvement of alkali tolerance in *M. nipponense*.

The measurement of antioxidant enzymes is a useful method to analyse the effects of behaviours of prawns after treatment with environmental stress [34, 35]. Many previous publications have been investigated the effects of alkali stress on antioxidant enzymes in plants [36–38], while the relative studies in aquatic animals were rare. The alkali concentration of pH 7.8 has positive effects on the activity of GPx, while strong alkalization (pH 8.8) inhibited the activities of antioxidant enzymes, suggesting high alkaline exposure negatively regulated the activities of antioxidant enzymes in the liver of hybrid tilapia [39]. High alkali treatment (32 mmol/L and 64 mmol/L) significantly stimulated the activities of SOD in the liver of *G. przewalskii* after 3 days of treatment [5]. Alkaline treatment stimulated the activities of SOD and CAT in the liver of *Triplophysa dalaica*, while they will then decrease to normal level [40]. In the present study, the alkali treatment stimulated the activities of MDA, GSH and GSH-PX in the gills of *M. nipponense*. The activities of MDA reached the peak at the alkali concentration of 12 mmol/L, while GSH and GSH-PX showed the highest activities at the alkali concentration of 8 mmol/L. These evidences indicated that MDA, GSH and GSH-PX are involved in the protection of the gills from the damage in *M. nipponense*, caused by alkali treatment.

Some previous publications have been identified the morphological changes of gills in aquatic animals after the alkali treatment. The *L. waleckii* with strong ability of alkali tolerance can maintain the normal structure and physiological functions of gills that can adapt the water environment with high alkali concentrations, while the phenomenon of fusion and detachment of gill cells were observed in the *L. waleckii* with low ability of alkali tolerance, causing loss of physiological function [41]. The morphological structures were observed to be changed in *G. przewalskii*, in order to adapt the water environment with high saline-alkali concentrations [42–44]. Alkali treatment leads to the deformation and fracture of gill fragments in *Eriocheir sinensis* [45]. In the present study, low alkali concentrations (< 4 mmol/L) did not result in the morphological changes of gills, while the treatment of high alkali concentrations (> 8 mmol/L) lead to the significantly morphological changes of gill membranes and haemolymph vessel, thus, affecting the normal respiratory function of gill in *M. nipponense*.

In the present study, the metabolic profiling analysis identified 171 DEMs between 0 mmol/L vs. 4 mmol/L, 37 DEMs between 0 mmol/L vs. 8 mmol/L, and 87 DEMs between 0 mmol/L vs. 12 mmol/L. Furthermore, the main enriched metabolic pathways of DEMs were identified as Metabolic pathways, Biosynthesis of secondary metabolites, Biosynthesis of plant secondary metabolites, Microbial metabolism in diverse environments, Biosynthesis of amino acids, which were consistent with the previous publications after the environmental stress in plants and aquatic animals [46–49].

Biosynthesis of secondary metabolites has been identified to positively regulate health. Secondary metabolites are natural products, which are necessary and synthesized by cells when cells stopped division. The analyses of both medicinal secondary metabolites and pharmacologically inactive secondary metabolites promote the understanding of the chemosynthetic functions of non-proliferating microbial cells [50, 51].

Microbial metabolism in diverse environments plays essential roles in adaption of the treatment of various environments in the wild organisms. Microbial metabolites have been identified to be involved in aerobic biodegradation, which are considered as indicators for rapid assessment of biochemical oxygen demand, due to their constituents depending on the oxidizing organic matter [52].

The transcriptome profiling analyses have been performed in several aquatic animals, which showed strong abilities of alkali tolerance, in order to select metabolic pathways and genes, involved in the process of alkali tolerance. These aquatic animals included *Lateolabrax maculatus* [10], *Luciobarbus capito* [11], and *Leuciscus waleckii* [13], and phenylalanine, tyrosine and tryptophan biosynthesis, cell cycle and DNA replication were identified as the main metabolic pathways, involved in the alkali tolerance in these aquatic animals. The present transcriptome profiling analysis revealed that a total of 1,107, 177, and 3,294 DEGs were identified between the comparison of 0 mmol/L vs. 4 mmol/L, 0 mmol/L vs. 8 mmol/L, and 0 mmol/L vs. 12 mmol/L, respectively, indicating high alkali concentration has stronger regulatory effects on the changes of gene expressions than those of the treatment with low alkali concentration in *M. nipponense*. In addition, a total of 235 DEGs (0 mmol/L vs. 4 mmol/L), 217 DEGs (4 mmol/L vs. 8 mmol/L), and 424 DEGs (8 mmol/L vs. 12 mmol/L) were annotated in KEGG database, and KEGG analysis revealed that Phagosome, Lysosome, Glycolysis/Gluconeogenesis, Purine Metabolism, Amino sugar and nucleotide sugar metabolism, and Endocytosis represented the main enriched metabolic pathways in all of these three comparisons.

Lysosomes are organelles, which contains many hydrolytic enzymes, and have biological functions to digest biological macromolecules, including proteins, nucleic acids, and polysaccharides [53–55]. Lysosomes play essential roles in the digestion of substances in cells which enter the cell from the outside, as well as to digest the local cytoplasm or organelles of the cell [56, 57]. Phagosome must exist in the bacteria as host, and can partially cause the lysis of host bacteria. Phagosome formation stimulates the production of reactive oxygen species (ROS) through the activation of the NADPH oxidase. It promoted the production of superoxide anion and ROS [58]. The new phagosome can quickly recognise the endosomes, and fuse with sorting and recycling endosomes [59]. Rab5 is required for the membrane fusion between the phagosome and endosomes requires, which is a membrane small GTPase. Phagosome maturation is normally accomplished by fusion with endosomal and lysosomal compartments, which occurs by a different mechanism in neutrophils. Phagosome maturation was generally happened with the loss of Rab5, and the presence of Rab7 [60]. Some DEGs were enriched in the metabolic pathways of both phagosome and lysosomes. Lysosome-associated membrane protein 1 (LAMP-1) is a glycoprotein, which is extensively expressed in lysosomal membranes. It serves as a barrier to prevent the liberation of soluble cathepsins and hydrolases into cytoplasm [61]. Cysteine proteinases is the most important type of endogenous proteinases, which is responsible for the process of autolysis process, including cathepsin and calpain [62]. Cathepsins are lysosomal proteases, which have various important biological functions in organisms, including regulation of cell death, degradation of intracellular and extracellular proteins, and activation of immune cells [63].

Glycolysis/Gluconeogenesis is identified as the key metabolic pathway to obtain energy in organisms. Gluconeogenesis catalysed the conversion of the non-sugar substances (such as certain amino acids, lactate, pyruvate, and glycerol) into glycogen or glucose during starvation and long-term muscle work under the catalysis of enzymes [64, 65]. Glycolysis promotes the degradation and metabolism of glycogen or glucose under anaerobic conditions to produce ATP, which is also a preparatory pathway for the aerobic oxidation of glucose in most organisms [66, 67]. Pyruvate kinase (PK) plays essential roles in catalysing the conversion of phosphoenolpyruvate (PEP) and ADP to pyruvate and ATP [68]. It is the final step in the process of glycolysis. PK plays essential roles in controlling the ATP concentration, indicating it has regulatory roles in maintaining the homeostasis of cellular energy [69, 70]. Phosphoenolpyruvate carboxykinase (PEPCK) plays a critical role in the conversion of the intermediate oxaloacetate from the tricarboxylic acid cycle into the gluconeogenic precursor phosphoenolpyruvate [71]. PEPCK also identified as a linkage between organic acid metabolism and nitrogen metabolism [72, 73].

The final products of purine metabolism are uric acid, which is converted from the xanthine by the catalysis of xanthine oxidase [74, 75]. Uric acid is a potent scavenger of ROS and RNS under the normal physiological conditions. The non-enzymatic oxidation of uric acid will occur, resulted in the generation of allantoin (5-ureidohydantoin) as a product, when an overproduction of ROS and RNS was happened. The disorders of purine metabolism resulted in a wide range of phenotypes, including neurologic abnormalities, hematologic abnormalities, and nephrolithiasis/gout [76, 77]. Guanylate cyclases (GCs) plays essential roles in catalysing the formation of guanosine 3’,5’-cyclic monophosphate (cGMP) from guanosine-5’-triphosphate (GTP). The soluble GCs and membrane-bound GCs were identified as the two main classes, which include the natriuretic peptide receptors [78–80].

The products of amino sugar and nucleotide sugar metabolism have many regulatory roles in living organisms, of which these products are important for maintaining and repairing the cell wall. Amino sugars and nucleotide sugars are important substances in living organisms, playing important roles in cells. Amino sugars is produced through glucose metabolism, which is an important material for energy metabolism. Amino sugars regulated many biological processes within cells, including the signal transduction and cell recognition [81, 82]. Nucleotide sugar is an important metabolic product, which is widely existed in cells. The metabolism of nucleotide sugar plays essential roles in the regulation of signal transduction, cell division, and cell apoptosis [83, 84]. Chitinases are known as a group of hydrolytic enzymes, which are synthesized by higher plants, insects and microbes (fungi, bacteria and viruses). Chitinase plays essential roles in the innate immune response, and its expression is upregulated under the environment with stress [85, 86]. Phosphoglucomutases are ubiquitous enzymes, which is widely existed in the living kingdoms. Phosphoglucomutase plays essential roles in the regulation of carbohydrate metabolism, catalysing the reversible conversion of 1- to 6-phosphoglucose [87, 88].

Endocytosis is a cellular process, which has been identified to regulate the process of cell signalling, nutrient uptake and the mediation of receptor internalization. Endocytosis regulates certain plasma-membrane proteins to be concentrated and efficiently internalized. This mechanism can internalize the transferrin receptor and low-density lipoprotein (LDL) receptor on cell-surface, promoting the binding of iron-loaded transferrin protein and cholesterol-bearing LDL particles with these receptors, and transferred into the cell. The endocytic vesicle usually fuses with the early endosome after endocytosis, accepting newly endocytosed material. The incoming proteins and lipids were then transferred to their final destination [89]. HSPs are a proteins family, which are extensively expressed by cells, in order to response the effects of stress. The previous publications have been identified the regulatory roles of Hsp70 in the resistance of external stress [90, 91]. Cellular homeostasis depends on pathways to selectively eliminate the aberrant and superfluous proteins, which have inhibitory effects on biochemical processes. E3 ubiquitin ligases plays essential roles in the specific recognition and degradation of eukaryotic proteins [92, 93].

qPCR analyses of DEGs were consistent with those of RNA-Seq, indicating the accuracy of the RNA-Seq. High alkali treatment (> 8 mmol/L) significantly stimulated the expressions of DEGs from the metabolic pathways of Phagosome and Lysosome, which also indicated the important roles of these metabolic pathways and the genes from these metabolic pathways in the process of alkali tolerance in *M. nipponense*. The expressions of *PEPCK*, *galU*, and *Dab* reached the peak at the alkali concentrations of 12 mmol/L, and the expressions of *PK*, *RGC*, *RRL*, Phosphoglucomutase, and *HSP70* reached the peak at the alkali concentrations of 8 mmol/L, predicting these genes were also involved in protection of body from the damage, caused by the alkali treatment.

In conclusion, the present study revealed that the alkali treatment stimulates the activities of MDA, GSH and GSH-PX, indicating these antioxidant enzymes plays essential roles in the protection of the body from the damage, caused by alkaline. In addition, high alkali concentration (> 8 mmol/L) leads to the damage of membrane and hemolymph vessel, thus affecting the normal respiratory function of gill. The metabolic profiling analysis identified that Metabolic pathways, Biosynthesis of secondary metabolites, Biosynthesis of plant secondary metabolites, Microbial metabolism in diverse environments, Biosynthesis of amino acids represent the main metabolic pathways of DEMs in the present study. The transcriptome profiling analysis revealed that a total of 1,107, 1,181, and 1,903 DEGs were identified between the comparison of 0 mmol/L vs. 4 mmol/L, 0 mmol/L vs. 8 mmol/L, and 0 mmol/L vs. 12 mmol/L, respectively. KEGG analysis revealed that Phagosome, Lysosome, Glycolysis/Gluconeogenesis, Purine Metabolism, Amino sugar and nucleotide, and Endocytosis were the main enriched metabolic pathways among all of these three comparisons. Phagosome, Lysosome, Amino sugar and nucleotide, and Endocytosis are immune-related metabolic pathways, while Glycolysis/Gluconeogenesis and Purine Metabolism were energy metabolism-related pathways. qPCR analysis verified that alkali treatment significantly stimulated the expressions of genes in the metabolic pathways of Phagosome and Lysosome. The present study carried out the effects of alkali treatment on gills of *M. nipponense*, providing valuable evidences for the genetic improvement of alkali tolerance in this species.

## Data Availability

The raw data of the present study have been submitted to NCBI with the accession number of SRX22743806-SRX22743817, and MetaboLights with the accession number of MTBLS9117.
